# Effect of mangosteen (*Garcinia mangostana L*.) peel extract as an antibiotic growth promoter on growth performance and antibiotic resistance in broilers

**DOI:** 10.14202/vetworld.2020.796-800

**Published:** 2020-04-27

**Authors:** Okti Herawati, Tri Untari, Marla Anggita, Sidna Artanto

**Affiliations:** Department of Microbiology, Faculty of Veterinary Medicine, Universitas Gadjah Mada, Yogyakarta, Indonesia

**Keywords:** antibiotic resistance, antibiotics, broiler, growth promoters, mangosteen

## Abstract

**Background and Aim:**

Antibiotic resistance poses a risk to human health and has therefore been the focus of research. One of the causes of this resistance is the use of antibiotics as feed additives for animal nutrition. The development of antibiotic resistance in poultry through nutrition feed has drawn attention to the need for alternative antibiotic growth promoters (AGPs). Mangosteen (*Garcinia mangostana L*.), as a natural source of bioactive phytochemicals, is a potential AGP, but the effect of mangosteen-based treatment on antibiotic resistance in poultry has not been reported to date. Therefore, the aim of this study was to evaluate the effects of mangosteen peel extract as an AGP on body weight gain, feed conversion rate (FCR), and the antibiotic resistance in broilers.

**Materials and Methods:**

In this study, 30 1-day-old broiler chicks were divided into three groups. Group A (control) was not administered any treatment in the feed, Group B was treated with 0.3 g/kg colistin as the AGP in the feed, and Group C was treated with 2% mangosteen peel extract as the AGP in the feed; the treatments were administered for 30 days. The observed parameters included the effect of the treatments on body weight gain, feed intake, FCR, and the presentation of antibiotic resistance before and after the treatments (pre-treatment and post-treatment, respectively).

**Results:**

Post-treatment, the body weight gain, and feed intake in the broilers were not significantly different among all the groups; however, the body weight gain and FCR were significantly different between the control group and the treatment groups in the 3rd week of treatment and were not significantly different between Groups B and C. The rate of antibiotic resistance to chloramphenicol increased significantly by 40% in Group B post-treatment, but no such increase was observed in Groups A and C.

**Conclusion:**

The findings of our study indicate that compared with using colistin as an AGP using mangosteen peel extract as a natural AGP did not have any significantly different effect on body weight gain, feed intake, and FCR (p>0.05) but had a significantly different effect on the rate of antibiotic resistance in broilers (p<0.05). This study indicates the usefulness of mangosteen for improving the overall growth and production performance of broilers without increasing their antibiotic resistance.

## Introduction

Antibiotic resistance is a serious global concern that has become a major challenge in human and animal research [1]. In the USA, bacterial strains with antibiotic resistance reportedly account for at least 2 million infection cases and 23,000 human deaths every year [2]. Antibiotic resistance has also been reported in Indonesia; the 2012 surveillance data obtained from the Ministry of Health reports showed that the prevalence rate of extended-spectrum β-lactamase-producing *Escherichia coli* had increased to 52% in Indonesia [3]. Antibiotic resistance is caused by various factors such as the excessive empirical administration of antibiotics in clinical practice, failure to complete the antibiotic dose as per the regimen, the indiscriminate use of antimicrobials in agriculture, and the rate and duration of using the antimicrobial therapy [4]. In the agricultural sector, particularly in the poultry industry, antibiotics have been used as feed additives to treat and prevent infections or to improve the growth and production performance of chickens [5].

Based on the data of the application of antibiotic growth promoters (AGPs) in animal production consist neomycin, erythromycin, oxytetracycline, streptomycin and colistin, particularly in chicken production, the global consumption of antimicrobials has been predicted to increase by 67% between 2010 and 2030 [6]. The increased use of AGPs in chickens potentially imposes an elevated selection pressure on bacteria to become resistant to antimicrobials [5,6].

The development of antibiotic resistance in chickens has drawn attention to the need for alternative AGPs. AGPs from natural sources could play a key role in improving the growth and production performance of chickens. One of the natural plant sources that can be used as AGPs is mangosteen (*Garcinia mangostana L*.) peel, which comprises bioactive phytochemicals such as xanthones, benzophenones, flavonoids, bioflavonoids, phenols, and triterpenes [7,8]. These bioactive phytochemicals function as an antibiotic and reportedly improve the growth and production performance of broilers, indicating the potential use of mangosteen as an AGP [9,10]. However, to the best of our knowledge, no study to date has elucidated the effect of using mangosteen peel as an AGP on the antibiotic resistance developed in broilers.

Therefore, the present study aimed to evaluate the effect of using mangosteen peel as an AGP in broiler feed on body weight gain, feed conversion rate (FCR), feed intake, and antibiotic resistance in broilers.

## Materials and Methods

### Ethical approval

All experiments of this research study were approved by the ethical committee of our institution (reference number: 00034/04/LPPT/VII/2019).

### Preparation of 2% mangosteen peel extract

The mangosteen peel extract was prepared, as described previously [11]. Briefly, mangosteen pericarps were sliced and dried. Then, ethanol (1000 ml) was added to 100 g of the mangosteen peel slices. The solution comprising these slices was filtered and heated in a rotary vacuum evaporator at 50°C. This solution was heated again in an oven at 90°C and filtered to obtain the final mangosteen peel extract. The solution was filtered with WhatMan No 1 filtered paper (24 cm). The filtrates were separately concentrated *in vacuo* using Rotary Evaporator (Model RE52A, China). The extract was stored in a refrigerator.

### Study period and study location

The study was conducted from June to August 2019 at Department of Microbiology, Faculty of Veterinary Medicine, Universitas Gadjah Mada, Yogyakarta, Indonesia.

### Experimental design

A total of 30 1-day-old broiler chicks (Cobb500) were divided into three groups. The chicks were kept for seven days to acclimatize. Within this period, they were fed commercial broiler starter diet only and given plain water. The number of 1-day-old chicks included in this study was calculated, according to Federer’s method [12]. Group A served as the control and was not administered any treatment in the feed, Group B was administered 0.3 g/kg colistin in the feed, and Group C was administered the prepared 2% mangosteen peel extract in the feed. The chicks were treated for 30 days. The growth performance parameters, including body weight gain, feed intake, FCR, and the presentation of antibiotic resistance, were evaluated in the broilers. The broilers were weighed every day to measure the body weight gain. Feed intake was recorded daily by measuring the amount of feed remnants. FCR was calculated by dividing the amount of feed intake with the body weight gain. The rate of antibiotic resistance (%) was calculated by dividing the number of bacteria that showed resistance with the total number of bacteria, as described previously [13,14]. The observations were recorded before (pre-treatment) and after (post-treatment) the treatments.

### Antibiotic sensitivity test

Eight standard antibiotic disks for ciprofloxacin, colistin, amoxicillin, streptomycin, chloramphenicol, tetracycline, ampicillin, and gentamicin were used against the isolated bacteria after the preparation of standardized bacterial inoculums matching the 0.5 McFarland standards (2×108 cfu/ml). The inoculum was spread on sterile Mueller-Hinton agar plates and incubated at 37°C for 24 h. The degree of sensitivity was determined by measuring the inhibition zone diameter, as described previously [15,16]. The results were interpreted according to the guidelines of the Clinical and Laboratory Standards Institute [16].

### Statistical analysis

The quantitative data from the different treatments in this study were statistically analyzed using analysis of variance (ANOVA), Kruskal–Wallis test, and marginal homogeneity test with p<0.05 were considered statistically significant.

## Results

### Body weight gain

Table-1 summarizes the changes in the body weight in terms of body weight gain in all the groups analyzed using ANOVA. The body weight of broilers increased post-treatment, but the body weight gain in the 1st, 2nd, and 4th weeks of treatment was not significantly different among the groups (p>0.05). The body weight gain in Group A (control) was significantly different from that in Groups B and C (treatment groups) in the 3rd week of treatment (p<0.05). Furthermore, the body weight gain was not significantly different between Groups B and C in the 3rd week of treatment.

**Table-1:** The effect of mangosteen peels extract on chicken body weight.

Parameters	Group A	Group B	Group C
Body weight			
Day 1	51.90±6.12	50.50±5.46	47.60±4.81
1^st^ week	445.50±46.898	453.80±47.138	460.80±38.378
2^nd^ week	719.40±68.850	718.00±78.330	667.20±49.620
3^rd^ week	1198.20±88.853	1220.70±145.223	1167.10±83.298
4^th^ week	1389.80±109.700	1393.80±212.756	1401.60±153.569
Body weight gain			
1^st^ week	393.60±43.436	403.30±44.535	413.20±37.371
2^nd^ week	273.90±31.370	264.20±37.199	206.40±36.127
3^rd^ week	478.80±104.303^a^	502.70±79.462^b^	499.90±49.298^b^
4^th^ week	191.60±152.030	173.10±108.575	234.50±184.117

a,b Different superscript indicates significant different. Group A=control, Group B=given AGP, Group C=given mangosteen peels extract (p<0.05). AGP=Antibiotic growth promoter

### Feed intake and FCR

The results of the Kruskal–Wallis test showed that all the groups did not show significant differences in feed intake (p>0.05), but the control and the treatment groups showed significant differences in FCR in the 3rd week (p<0.05) (Table-2). However, FCR was not significantly different between Groups B and C in the 3rd week of treatment.

**Table-2:** The effect of mangosteen peels extract on feed intake and FCR.

Parameters	Group A	Group B	Group C
Feed intake			
1^st^ week	175.80±0.0032	164.20±0.0030	198.80±0.0029
2^nd^ week	261.93±0.0033	261.26±0.0029	237.06±0.0028
3^rd^ week	466.66±0.0030	466.67±0.0028	466.65±0.0020
4^th^ week	786.66±0.0025	786.65±0.0205	786.66±0.0029
FCR			
1^st^ week	0.398±0.038	0.412±0.046	0.485±0.045
2^nd^ week	0.366±0.033	0.367±0.038	0.357±0.026
3^rd^ week	1.057±0.431^a^	0.386±0.043^b^	0.401±0.028^b^
4^th^ week	0.569±0.042	0.576±0.088	0.554±0.059

a,b Different superscript indicates significant different. Group A=control, Group B=given AGP, Group C=given mangosteen peels extract (p<0.05). FCR=Feed consumption rate. AGP=Antibiotic growth promoter, FCR=Feed conversion rate

### Antibiotic resistance findings

The marginal homogeneity test showed that the broilers who were treated with colistin as an AGP (Group B) showed a significant increase in the rate of antibiotic resistance to chloramphenicol (p<0.05) between pre-treatment and post-treatment. However, the broilers who were treated with 2% mangosteen peel extract (Group C) did not show any increase in the rate of antibiotic resistance (Table-3).

**Table-3:** Effect of mangosteen peels extract on percentage of antibiotic resistance (%).

Antibiotic	Group	Pre-treatment	Post-treatment
CIP	A	100	100
	B	100	100
	C	100	100
CT	A	100	80
	B	100	90
	C	100	90
AML	A	100	100
	B	100	100
	C	100	100
C	A	70	90
	B	50^a^	90^b^
	C	100	80
S	A	100	100
	B	90	100
	C	100	100
TE	A	100	100
	B	100	100
	C	100	100
AMP	A	100	100
	B	100	100
	C	100	100
CN	A	90	100
	B	90	100
	C	100	100

a,b Different superscript indicates significant difference. Group A=control, Group B=given AGP, Group C=given mangosteen peels extract. CIP=Ciprofloxacin, CT=Colistin, AML=Amoxicilin, C=Cloramphenicol, S=Streptomycin, TE=Tetracycline, AMP=Ampicilin, CN=Gentamicin (p<0.05). AGP=Antibiotic growth promoter

## Discussion

The present study analyzed the effects of adding mangosteen peel extract to broiler feed on body weight gain, feed intake, FCR, and the presentation of antibiotic resistance. Colistin is widely used as a growth promoter in poultry production, but with the increasing incidence of antimicrobial resistance, natural sources such as mangosteen have been explored for their potential as AGPs. We found that the control and treatment groups showed significantly different body weight gain in the 3rd week of treatment. This finding is similar to that reported by a previous study [17], wherein the use of AGPs as feed additives demonstrated improved growth and production performance in 4 weeks as well as efficiency. Antibiotics administered at subtherapeutic doses are expected to reduce the number of pathogenic microorganisms in the digestive tract to ensure that the poultry is healthy and can utilize all the nutrients in the feed, with a rapid growth rate and enhanced production efficiency [18]. In the present study, AGP didn’t affected to body weight gain in the 1^st^, 2^nd^, and 4^th^ weeks of treatment; therefore, their nutritional efficiency was considered low because of the pathogenic microorganisms that remained in the digestive tract. This finding is similar to that reported by a previous study [19] stating that antibiotic supplementation is not associated with body weight gain and microorganism types in the intestine. Therefore, the FCR in the control and treatment groups in the present study was also not found to be significantly different (p>0.05).

The rate of antibiotic resistance to ciprofloxacin, colistin, amoxicillin, streptomycin, tetracycline, ampicillin, and gentamicin pre-treatment and post-treatment was not significantly different (p>0.05) among all the groups. However, the rate of antibiotic resistance to chloramphenicol in Group B (colistin-treated group) was significantly different (p<0.05) from that in Groups A and C. The rate of resistance to chloramphenicol increased by 40% after the administration of colistin. This finding is consistent with that reported by a previous study [15], wherein the rate of antibiotic resistance increased by 3-70% after supplementation with additional AGPs in animal feed.

Antibiotic resistance can occur through various mechanisms such as antibiotic inactivation, membrane permeability reduction, antibiotic target modification, and antibiotic transport modification [20]. Furthermore, antibiotic inactivation can occur when microorganisms produce enzymes such as beta-lactamases that hydrolyze the beta-lactam ring, consequently inhibiting the function of beta-lactam antibiotics, for example, beta-lactamase destroys beta-lactam from penicillin, which inhibits antibiotics from adhering to the peptidoglycan of bacterial wall and eventually leads to inhibition of antibiotic function [20,21]. Changes in bacterial cell membrane permeability can occur due to genetic mutations that cause pore changes in the bacterial cell wall so that antibiotics cannot enter the bacterial cell [21]. Modification of antibiotic targets can cause antibiotic resistance because changes in the molecular structure of the antibiotic target can result in the failure of antibiotics to bind to the target, thereby making the antibiotic ineffective [22]. Another antibiotic resistance mechanism involves the removal or transportation of antibiotics such as macrolides, tetracyclines, and fluoroquinolones outside the bacterial cells, thereby making these antibiotics ineffective against bacterial infections [23].

In this study, the rate of antibiotic resistance to chloramphenicol was found to be increased in the treatment group with colistin added as an AGP. According to a previous study [24], the administration of colistin causes cross-resistance to other antimicrobials. This mechanism of cross-resistance may have caused a significant increase in the antibiotic resistance to chloramphenicol in the present study. In the present study, there was no increase in the resistance to other antibiotics because the rate of antibiotic resistance to ciprofloxacin, colistin, amoxicillin, streptomycin, tetracycline, and ampicillin in 1-day-old broiler chicks was already 100% pre-treatment and supplementation with colistin in the feed did not result in any further significant difference. However, the rate of antibiotic resistance to other antibiotics, namely, chloramphenicol and gentamicin, was <100% pre-treatment. These results are in accordance with those reported previously by Moreno *et al*. [25], who showed that 1-day-old chicks exhibit varying rates of antibiotic resistance (ranging from 5% to 75%) to different antibiotics. A similar result has also been reported by a previous study [26], wherein *E. coli* isolated from 1-day-old chicks showed resistance to penicillin and ciprofloxacin. A previous study [27] has shown that the rate of antibiotic resistance in 1-day-old chicks was much higher than that in the environment. This result was supported by Okorafor *et al*. [28], who showed that *E. coli* isolated from 1-day-old chicks is resistant to antibiotics, including tetracycline, ampicillin, gentamicin, enrofloxacin, ciprofloxacin, and streptomycin, with rates of antibiotic resistance reaching >80%.

As stated previously [29], the presentation of antibiotic resistance in 1-day-old broiler chicks can be due to either intrinsic resistance or acquisition resistance. Intrinsic resistance is caused by genetic mutations that occur in bacterial chromosomes, whereas acquisition resistance is caused by the transfer of resistant genes from the environment or by horizontal transfer from other bacteria [30,31].

## Conclusion

The findings of our study suggest that compared with colistin, mangosteen peel extract used as an AGP did not have any significantly different effects on the body weight gain and FCR of broilers. However, the mangosteen peel extract significantly affected the antibiotic resistance of broilers. While the colistin-treated group showed a significant increase in the rate of antibiotic resistance to chloramphenicol post-treatment, no such increase was observed in the mangosteen peel extract-treated group, indicating significant differences in the rate of antibiotic resistance between these two groups. Therefore, mangosteen peel extract can be considered a useful natural AGP that can promote the growth and production performance of broilers without increasing the rate of antibiotic resistance.

## Authors’ Contributions

OH and TU designed and managed the study. OH and MA collected and analyzed samples. SA, OH, TU, and MA arranged, analyzed, and wrote the manuscript. All authors have read and approved the final manuscript.
